# Validation of a Mobile Health Technology Platform (FeverTracker) for Malaria Surveillance in India: Development and Usability Study

**DOI:** 10.2196/28951

**Published:** 2021-11-10

**Authors:** Ipsita Pal Bhowmick, Dibyajyoti Chutia, Avinash Chouhan, Nilay Nishant, P L N Raju, Kanwar Narain, Harpreet Kaur, Rocky Pebam, Jayanta Debnath, Rabindra Tripura, Kongkona Gogoi, Suman Ch Nag, Aatreyee Nath, Debabrata Tripathy, Jotish Debbarma, Nirapada Das, Ujjwal Sarkar, Rislyn Debbarma, Rajashree Roy, Bishal Debnath, Dipanjan Dasgupta, Suraj Debbarma, Kamal Joy Tripura, Guneram Reang, Amit Sharma, Manju Rahi, Jyoti Chhibber-Goel

**Affiliations:** 1 Regional Medical Research Centre-Northeastern Region Indian Council of Medical Research Dibrugarh India; 2 North Eastern Space Applications Centre Umaim India; 3 Indian Council of Medical Research Delhi India; 4 National Institute of Malaria Research Indian Council of Medical Research Dwarka, Delhi India; 5 International Centre for Genetic Engineering and Biotechnology New Delhi India

**Keywords:** fever, health system, mHealth app, malaria, surveillance, mobile phone

## Abstract

**Background:**

A surveillance system is the foundation for disease prevention and control. Malaria surveillance is crucial for tracking regional and temporal patterns in disease incidence, assisting in recorded details, timely reporting, and frequency of analysis.

**Objective:**

In this study, we aim to develop an integrated surveillance graphical app called FeverTracker, which has been designed to assist the community and health care workers in digital surveillance and thereby contribute toward malaria control and elimination.

**Methods:**

FeverTracker uses a geographic information system and is linked to a web app with automated data digitization, SMS text messaging, and advisory instructions, thereby allowing immediate notification of individual cases to district and state health authorities in real time.

**Results:**

The use of FeverTracker for malaria surveillance is evident, given the archaic paper-based surveillance tools used currently. The use of the app in 19 tribal villages of the Dhalai district in Tripura, India, assisted in the surveillance of 1880 suspected malaria patients and confirmed malaria infection in 93.4% (114/122; *Plasmodium falciparum*), 4.9% (6/122; *P vivax*), and 1.6% (2/122; *P falciparum/P vivax* mixed infection) of cases. Digital tools such as FeverTracker will be critical in integrating disease surveillance, and they offer instant data digitization for downstream processing.

**Conclusions:**

The use of this technology in health care and research will strengthen the ongoing efforts to eliminate malaria. Moreover, FeverTracker provides a modifiable template for deployment in other disease systems.

## Introduction

### Background

Surveillance is defined as “the continuous and systematic collection, analysis, and interpretation of disease-specific data, and the use of that data in the planning, implementation, and evaluation of public health practice” [1]. Surveillance of malaria cases assists the health ministry to (1) calculate disease burden, (2) monitor changing disease patterns, (3) design effective health interventions, and (4) evaluate the impact of malaria control programs, thereby curtailing transmission [2]. Nationally coordinated strategies intensify control efforts directed toward populations with the highest disease transmission by means of targeted intervention [2,3]. Nations with weak surveillance systems fail to assess disease trends and plan interventions, thereby resulting in continued transmission of malaria. The 2016-2030 Global Technological Strategy for Malaria of the World Health Organization (WHO) encourages national malaria surveillance programs to accelerate progress from malaria control to elimination [4,5]. Surveillance systems comprise the community, health workers, tools, and structures required to generate, analyze, and interpret data. Subsequently, decision-makers use the data for planning and targeting interventions. A combination of effective activities such as passive case detection (PCD; ie, febrile patients reach out to health facilities and get diagnosed) and active case detection (ACD; ie, patients with malaria are detected by health workers through active search in the community) are used by the surveillance systems [6]. Health care workers engage in malaria surveillance via ACD and PCD at all levels of the health system and communicate data from the field to the district and state health levels. This is the usual channel of data communication deployed in malaria-endemic regions for malaria control and elimination.

Despite established surveillance strategies, malaria-monitoring technologies (eg, rapid diagnostic tests [RDT], data capture on parasites and vector drug or insecticide resistance, and disease transmission markers) in many countries remain insufficient to support targets of malaria elimination [7-9]. The National Vector Borne Disease Control Programme (NVBDCP) of India is the largest surveillance network in the world [2]. Despite a well-defined active and passive surveillance system and a decreasing trend in malaria cases, gross discrepancies between reported malaria incidence in India, and the estimated malaria cases in the WHO published World Malaria Report have been noted [10,11]. A recent study estimated malaria incidence to be four-fold greater than one million reported by the NVBDCP but three-fold less than 13 million estimated by the WHO, whereas the estimated deaths were 93-fold more than the average 313 deaths reported by the national malaria program in 2015-2016 [12]. NVBDCP surveillance misses malaria cases diagnosed and treated by private and other health sectors; for instance, those operating in remote areas must be captured to determine the true malaria burden [13]. In addition to the significant gap between the reported and estimated malaria cases and deaths, in a country with approximately 1.3 billion people, delayed communication of the data from the grassroots-level health workers (especially in remote areas) to the district and further up to the state level results in the lack of prompt data compilation and analysis and hence timely and appropriate mitigation action. The challenge lies in the switch from aggregated, paper-based reporting systems to near real-time, case-based electronic systems [14]. Furthermore, malaria transmission is not homogenously distributed either geographically or within populations. The WHO emphasizes the need to identify malaria transmission foci and *hotspots* within endemic regions [1]. Failure to identify hotspots would result in sustained residual malaria transmission, thus undermining malaria control programs.

### Objectives

The use of digital technology via the implementation of smartphone surveillance apps by the community for self-reporting and setting up digital information and contact chains at all levels of health care would be beneficial for effective malaria surveillance [1,13,14]. This avenue is equally applicable in urban and rural areas.

The worldwide smartphone internet traffic accounts for approximately 51% of the total global web-based traffic, wherein smartphone-based internet use is often a major way to access information worldwide [15]. Furthermore, mobile devices (excluding tablets) account for approximately 80% of the total internet traffic in India, with approximately 448 million smartphone users as of December 2020 [15]. Here, we report the establishment of a new smartphone mobile health app called *FeverTracker*. The app connects the information provided by the user to the nearest health care center, thereby allowing the health care staff to reach symptomatic patients and perform malaria RDT and slides as required. Validation of the app in 19 malaria-endemic tribal villages situated in remote, forested hilly regions in Dhalai district of Tripura state for a period of 20 months was performed. The app use resulted in a shortening of time between the collection of data and its reporting by a month. The app has served to monitor malaria in an integrated fashion, giving an all-in-one tool to the 19 health care workers involved in malaria surveillance. To date, the app has been used to screen 1880 suspected patients and was able to detect 122 malaria cases (114/122, 93.4% *Plasmodium falciparum*; 6/122, 4.9% *P vivax*; and 2/122, 1.6% *P falciparum/P vivax* mixed infection cases). The use of the FeverTracker app aims to strengthen malaria surveillance and provide instant digitization of epidemiological data.

## Methods

### App Development

FeverTracker is based on the concept of a progressive web app using the Ionic (Drifty) and Cordova (developed by Joe Bowser, Michael Brooks and Team) platforms. The app can capture ground information on malaria incidents attributed to geographic positional information, geotagged photos, and other relevant data as per the prescribed format. FeverTracker supports multilingual data to cater to local requirements and inclusive use. Detailed symptom information and use cases are included in the app. Advisory regarding drug dose information based on user symptoms is also included. The app currently supports Android OS 4.4 (Google) or higher. A hypertext preprocessor (PHP; developed by Rasmus Lerdorf) webserver was used to deploy the app, and this app works on client-server architecture where in the backend, PHP and MySQL servers (Oracle Corporation) are used. The app also works in an offline mode, where the SQLite database (developed by D Richard Hipp) is used for record management. All communication between the mobile app and the backend server is secured using HTTP secure (European Organization for Nuclear Research). The mobile app is interlinked with an interactive web portal for data visualization, summaries, and data downloads. The app adheres to privacy laws and policies while seeking permission to access location and permission to use text messages, mobile data, and local storage. In addition to automated data capture, FeverTracker is linked to SMS text messaging and an advisory instruction system to notify the district or state response center according to national guidelines.

### Portal Development

The FeverTracker portal is built on top of an open-source stack and comprises major functionalities such as on-the-fly analytics, integration with mobile apps, visualization of rich and interactive geographic information system (GIS) maps, information box, print maps with statistics, and data updates. The app comprises a 3-tier architecture: (1) database, (2) GIS server, and (3) web client mapping. The architectural design follows a service-oriented architecture, where tier 1 consists of a database layer hosting Postgres (developed by Michael Stonebraker and team) or the MySQL database, wherein all the location information along with attributes are stored. In tier 2, the GIS server in use is geo-server, and the reporting tool used is map fish print (OSGeo project). The geospatial data are served via a geo-server and follow the open geospatial consortium standards, where the data serve as web mapping services as well as web feature services. The client side uses leaflet (developed by Vladimir Agafonkin) as a map display tool and D3js-based customization (developed by Mike Bostock, Jason Davies, Jeffrey Heer, and Vadim Ogievetsky) for querying across the parameters. The apps are deployed with a leaflet in the frontend and backend as PHP.

### Deployment of the App

The app was deployed in 19 villages of the Dhalai district of the state of Tripura in India, involving 19 health care workers that included 2 multipurpose workers (MPWs) posted at Gurudhan and Shikaribari subcenters and 3 Accredited Social Health Activists (ASHAs) who oversee 6 villages under these subcenters. In addition, 14 village volunteers designated for malaria surveillance in these 19 villages trained jointly by state and project were recruited for the deployment of the app. Fever surveillance in the community (ACD) or cases reported at subcenters (PCD) as well as seasonal mass surveillance undertaken by the state in the area was performed using the app. The health care workers using the app were given mobile handsets and power banks as backups because of frequent power cuts in these remote villages. A demonstration and hands-on training were provided to all app users.

### Survey for App Users

A survey using Google Forms was conducted among the 19 health care workers using the app for suspected malaria patient surveillance. The survey questions were in 2 local languages (1) Bengali and (2) Kokborok. The answers were translated into English.

### Preparation of Ecological Maps

The land use land cover mapping was prepared using the orthorectified Indian remote sensing satellite data, Cartosat-1 (2.5 m) and Linear Imaging Self Scanning Sensor–IV (5.8 m). On-screen visual interpretation techniques were used on a GIS platform. Major land use and land cover categories along with subcategories were delineated and updated using the latest data (2019) on the spatial layer. The spatial layer was prepared under National Remote Sensing Centre/Indian Space Research Organization’s space-based information support for decentralized planning at the panchayat level. Mapping was performed at 1:10,000 scales. The geolocations obtained from the FeverTracker app from villages screened under the Gurudhan and Shikaribari subcenters of Ambassa and Ganganagar were plotted on these ecological maps.

## Results

### Multifaceted Surveillance

**Figure 1:**
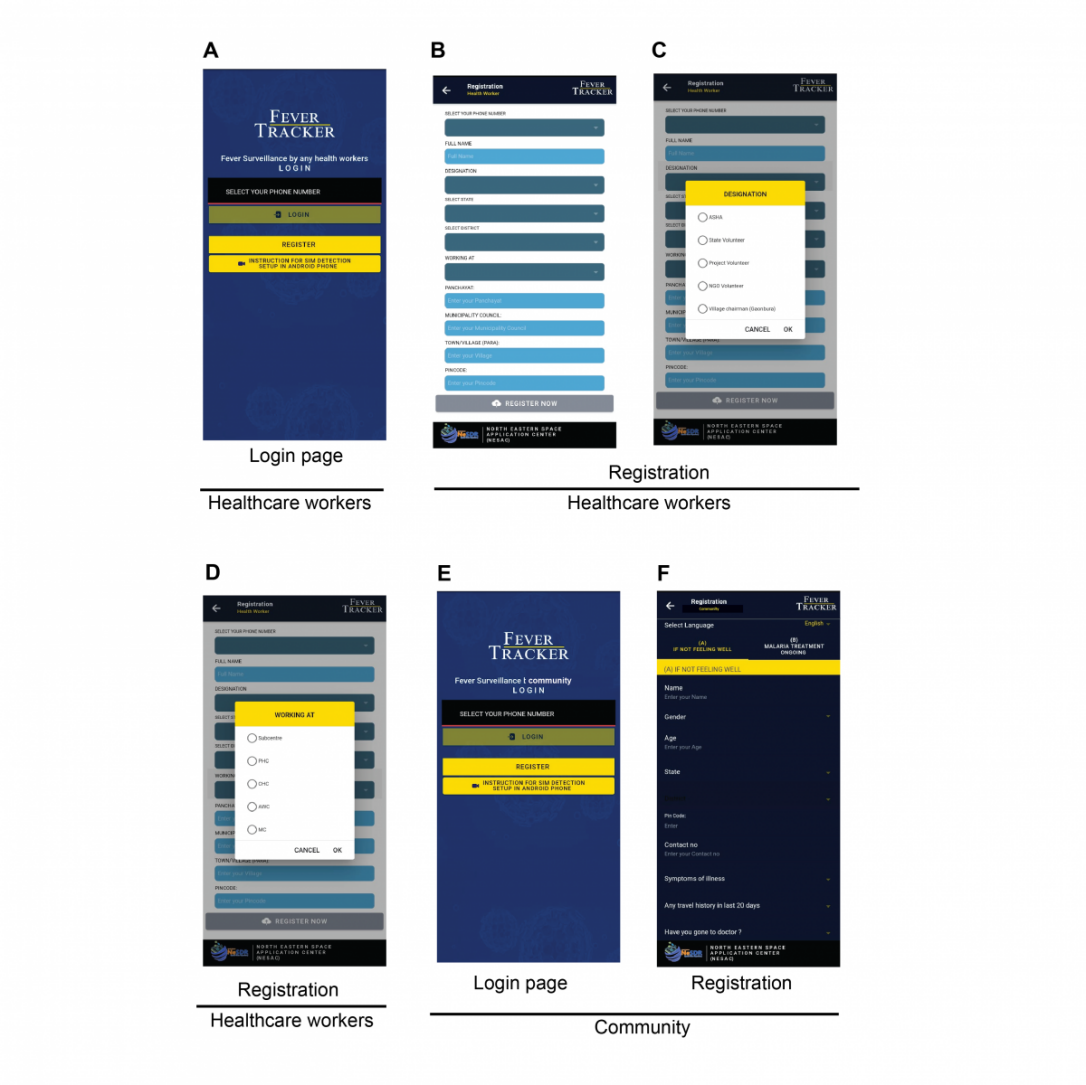
The log-in and registration page of the FeverTracker mobile health app. The community (A) log-in page and (B) registration page with required contact details, symptoms, travel history, and physician visit is shown. (C-F) The app allows the health care workers to log-in and register with their credentials and register, allowing them to perform surveillance at the grassroots level. Names of the persons and the villages have been removed to retain privacy.

The registered health care workers involved in door-to-door screening (ACD protocol) for malaria surveillance can use this app (Figure 1A). The app allows health care workers to register via their phone number and create a unique ID for future log-ins (Figure 1B). The list of health care workers involved is as follows: ASHAs, auxiliary nurse midwife, MPWs, community health officers at the field level and village volunteers, laboratory technicians, and medical officers engaged in testing and interpreting the results (Figure 1C). Notably, the app has the provision for private practitioners, laboratories, pharmaceuticals, and security forces to enter the data. The different levels of data collection and transmission will work for fever screening in the villages and at the subcenter, primary health centers, block level, district hospitals, or clinics (Figure 1D).

In addition, the app has provision for the participation of community via self-assessment and then to report malaria symptoms to health care workers, which will facilitate prompt health seeking and thus improve surveillance and malaria control. FeverTracker has features to assist the community in understanding their fever-related symptoms and to report them to health authorities [3]. This will supplement PCD with people self-reporting through the app and hence enhance surveillance with the help of at-risk communities. The app allows the patients screened for malaria via the private sector to be added as well as tracked for the accuracy of their treatment regimen, adherence, and compliance to the malaria drugs. In case of nonmalarial diseases, the individuals can be tested for other febrile illnesses. Further, based on their symptoms clustering, the outbreak of different diseases can be assessed and predicted. FeverTracker allows users to enter their details and register via cell phone numbers that are stored in the health center database in a systematic and traceable format (Figure 1E and F). The app further connects the information provided by the user to the nearest malaria surveillance center. On receiving the information on a suspected malaria case in their surveillance region, health care workers can take appropriate action, for example, perform rapid diagnostics and slide microscopy to confirm malaria infection. In the case of a positive test result, health care workers are notified, and malaria treatment is provided. This key feature is further discussed and elaborated in later sections. In addition, if the user has been tested and treated, the app allows the user to report these details along with the complete course of treatment.

**Figure 2:**
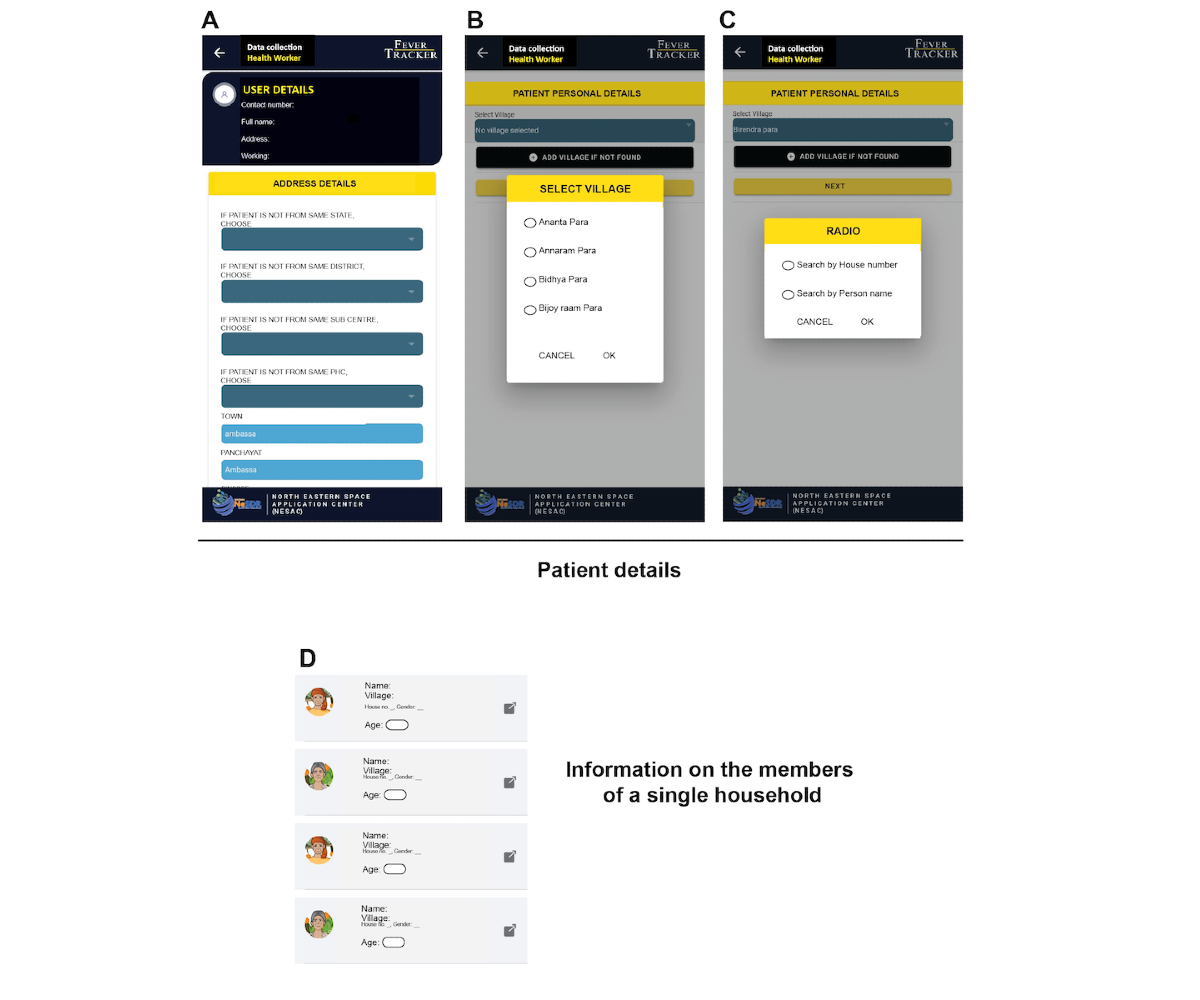
Data collection by health care workers using FeverTracker. (A-C) The contact details of the individual being surveyed or in case of a self-reported case are entered or verified by the health care workers. (D) The information on multiple family members of a single household is displayed on the app for the ease of health care workers during data collection and sampling. Names of the persons and the villages have been removed to retain privacy.

**Figure 3:**
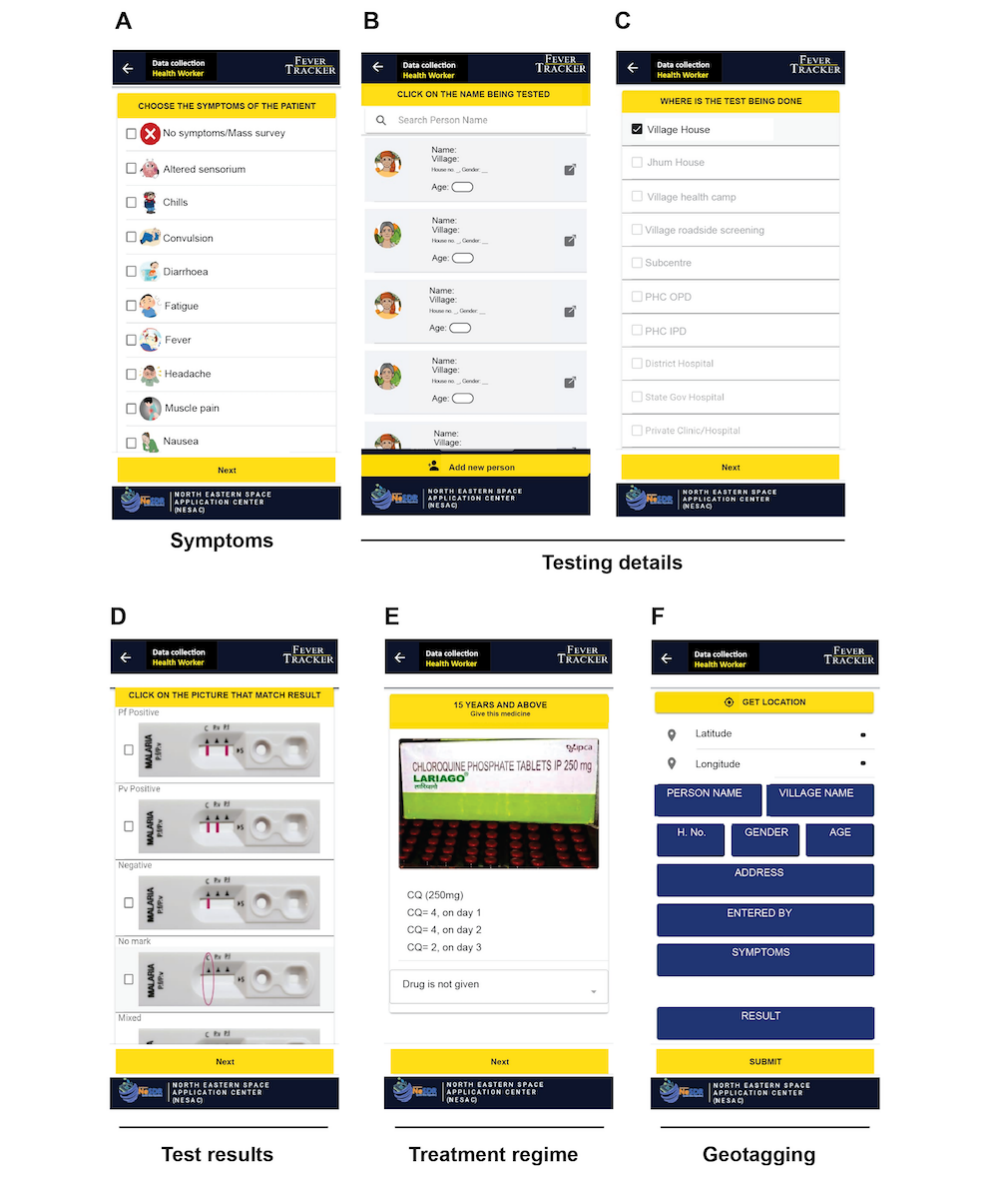
The key features of the surveillance performed using the FeverTracker. (A) The health care workers and community members, on registration, can choose the disease symptoms. The testing details including (B) the name of the individual being tested, (C) the location of the test, (D) the test results, and (E) the medicine prescribed are shown. The app includes geolocation information to assist health authorities in data visualization. Names of the persons and the villages have been removed to retain privacy.

Thus, the app allows both the community and health care workers to integrate in ACD and PCD fever surveillance. Each can enlist their address and details, including name, age, address, phone number, and upload a photograph (optional; Figure 2A-C). The details of the household members filled under the personal and address detail columns are shown in Figure 2D. At the time of data collection, information on the symptoms of the patients is entered by the health care workers or by the community when self-reporting (Figure 3A). In addition, if multiple individuals from a single household were tested, the details of each household member are entered (Figure 3B) along with the location of the testing center or place (Figure 3C). To ensure that this process is smooth, accurate, and not tedious for health care workers, pictorial options are provided to select a wide range of symptoms in malaria and other vector-borne diseases, as well as the RDT result outcome (Figure 3A and D) and the treatment regime to be followed by the patients who test positive for malaria according to the national guidelines (Figure 3E).

### Offline Data Capture and Geomapping

For effective surveillance in remote parts of India with limited or no access to the mobile network, the FeverTracker app is functional offline. The app provides an offline data entry feature, wherein data remain stored on the device and are uploaded to the server when the network is available. Each data entry on FeverTracker includes geolocation information (Figure 3F). Geomapping assists health authorities in visualizing district and state-wide data to locate malaria clusters and regions that require urgent attention. Furthermore, records of individuals who previously tested positive for malaria are available to state health officials to help assist and track the travel history of malaria cases across states within the country. Currently, the app is operational in 19 tribal villages in the Dhalai district of the state of Tripura, India.

### Real-Time Data Capture and Coordinated Digitization of Surveillance

**Figure 4:**
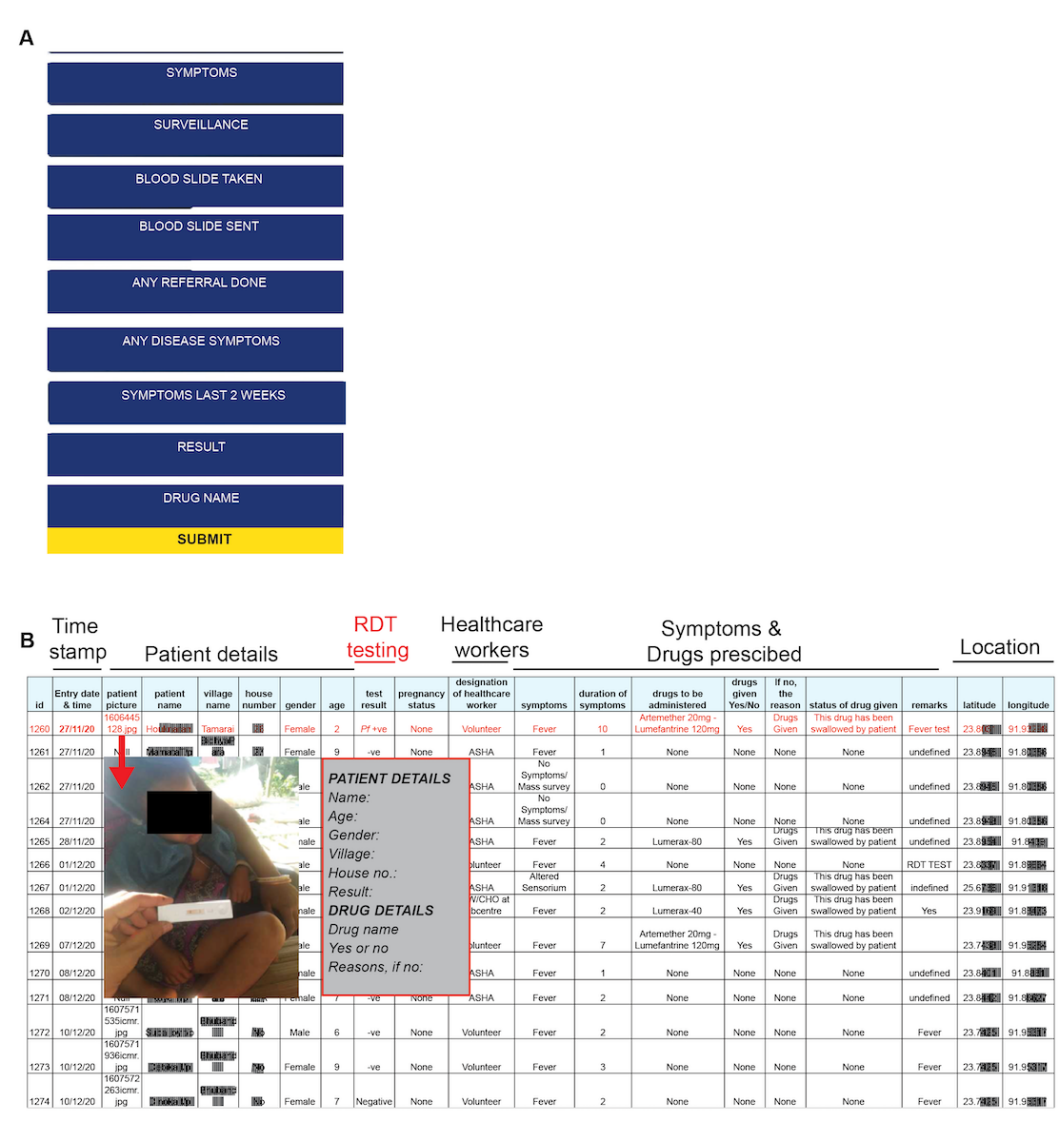
The deployment of the FeverTracker. (A) The malaria surveillance data collected as visualized within the app is shown. (B) The data transmitted and stored in the server for visualization are shown. The data are digitized via the web platform in a presentable format with patient details, rapid diagnostic test results, symptoms, and drugs prescribed along with geolocations. The screenshot of the SMS text message received by the health care worker on verification of data is shown. One patient data entry is highlighted in the data spreadsheet along with the corresponding SMS text message and the patient image. RDT: rapid diagnostic test.

Digitization of data is an important and indispensable tool for surveillance programs to manage, visualize, and analyze data collected for any disease. The data capture constitutes multiple steps and involves health care workers from different strata, such as (1) a data entry operator or data manager who can enter the data obtained from the health workers, (2) data from primary health care centers that are sent to block level and then collated for district-level compilation and analysis, and (3) reports compiled or generated are sent from the districts to the state surveillance teams. The data generated via the above surveillance are the basis for the implementation of policies, strategies, and operational activities. Incomplete data or delays in data communication prolong malaria control action. In this scenario, our app allows the community to participate in malaria surveillance at the grassroots level. Health care workers receive an update on the information input by the community, which allows them to focus on individuals and areas requiring immediate attention. At the time of submission of the data collected via the FeverTracker app, the following information is available to the health authorities for further action: (1) symptoms; (2) surveillance mode (ACD, PCD, or mass surveillance); (3) blood smear details; (4) duration of symptoms; (5) RDT test results, if any; and (6) medicine prescribed (Figure 4A). The surveillance team can edit and visualize the data on their mobile apps and on the web application dashboard (Figure 4B). After verification, the data entered into the app are communicated via an SMS text message to the concerned health care personnel and authorities. These data include test results with patient details and treatment status, thus allowing quick communication. The data from the FeverTracker main system are automatically digitized and made available via data sheets that can be used by the concerned district and state health workers and officials. Figure 4B shows the image of the data sheet with a patient entry highlighted along with the corresponding SMS text message and image of the patient with a date and time stamp to demonstrate the accuracy and usefulness of the app. The uploaded data can also be monitored by health officials via a web application platform in real time, allowing them to filter the data based on the set criteria for further analysis of malaria hotspots. In brief, the data input by the community and the data captured by health care workers are digitized in near real time and linked to the health centers via the SMS text message and advisory instructions system to notify the district or state response center.

### Backend Data Capture and Display

The mobile app–linked web application is equipped with analytical and mapping tools based on the data received from the app. The landing page has the following features for semiautomated live data streaming: (1) daily confirmed and cumulative cases spanning the current, recovered, and malaria deaths across the surveyed regions; (2) a GIS map based on the data captured via the FeverTracker app to identify high caseload zones showing malaria clusters and regions; (3) a map showing the malaria incidence for various districts; and (4) statistical analysis of the samples tested for malaria along with age- and gender-wise information. As a proof of concept, the data collected from the app are shown in Figure 4. The information for each patient is visible on the app, as shown in Figure 4A. This information is linked to the web application to be viewed in an Excel format as raw data (Figure 4B). The additional feature in the FeverTracker app linking it to the web application allows it to be equipped with analytical and mapping tools based on the data received.

### Data Collection on App Use to Understand Users’ Perspective

**Figure 5:**
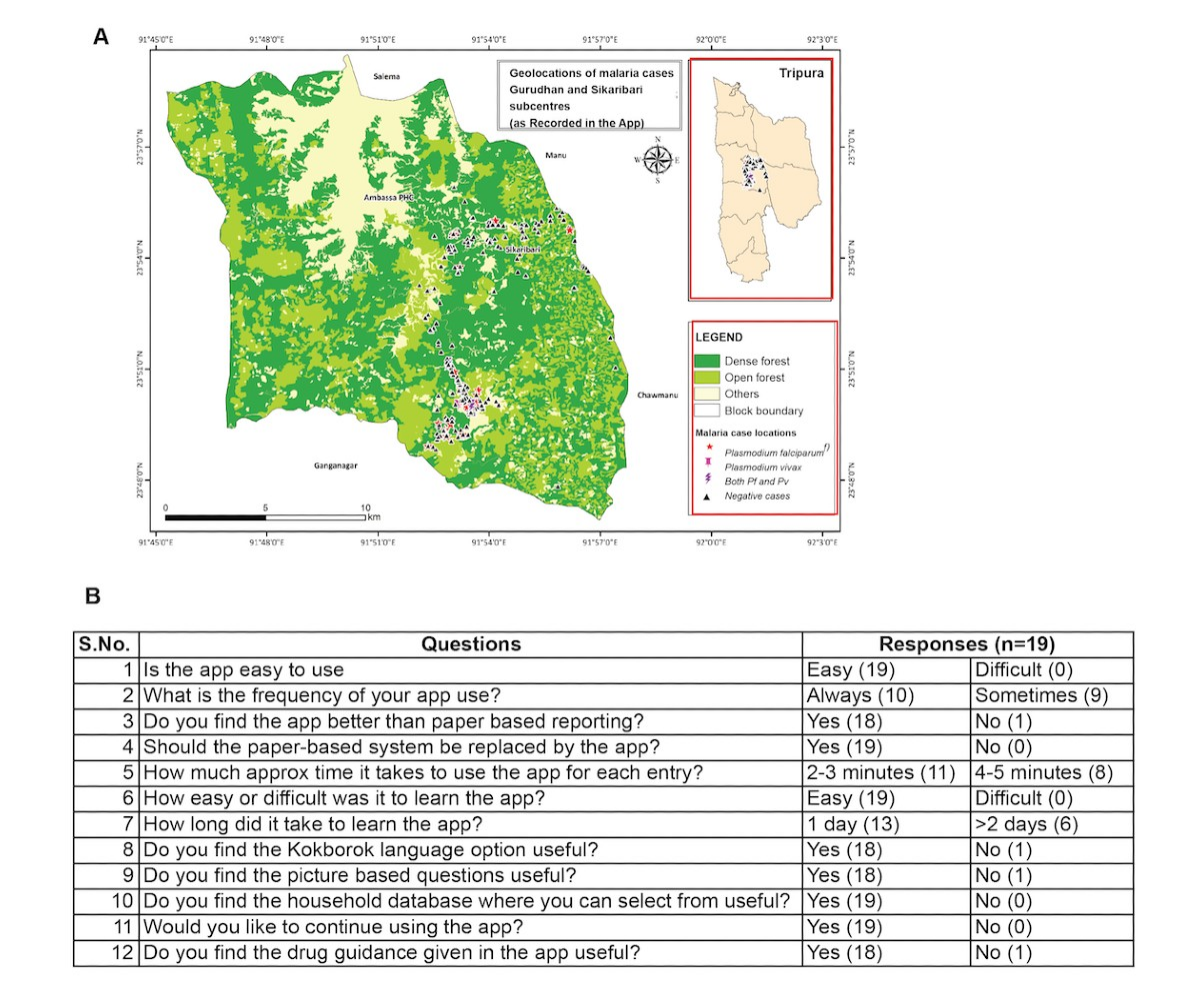
Population surveyed and the survey for the health care workers. (A) The geolocations of the suspected malaria cases tested in Gurudhan and Shikaribari subcenter villages under Ambassa block in the district Dhalai, Tripura, obtained through the FeverTracker app are shown. The surveyed patient data were plotted in the land cover mapping of Ambassa block that was prepared using the orthorectified Indian remote sensing satellite data, Cartosat-1 (2.5 m) and Linear Imaging Self Scanning Sensor–IV (5.8 m), using on-screen visual interpretation techniques in the geographic information system platform. The mapping was done at 1:10,000 scales. The map of the state of Tripura with the district boundaries is shown in the inset. Data on the malaria cases from the mentioned area as registered via the app are marked (negative cases [blue], *Pf* [red], *Pv* [pink], and *Pf/Pv* [purple]). (B) The details of the survey conducted are given. The questions asked and the replies from the 19 health care workers obtained with the number of responses (in bracket) are listed.

The app was deployed in 19 malaria-endemic villages under Gurudhan, Shikaribari, Karnamani, and Maldapara subcenters of Ambassa and Ganganagar Primary Health Centre in Dhalai district of Tripura, India, for 20 months. These 19 health care workers (MPWs, ASHAs, and village volunteers) used the app to survey and screen a population of 4870 and perform RDT on 1880 people with fever suspected of malaria. Among these patients, 122 tested positive for malaria infection (114/122, 93.4% *P falciparum* monoinfection; 6/122, 4.9% *P vivax* monoinfection; and 2/122, 1.6% *P falciparum*/*P vivax* mixed infection cases), and the treatment provided was recorded. As part of this study, the health care workers involved in testing the app collated information on the symptoms, testing, treatment regime, and geolocations of suspected and positive patients with malaria. Figure 5A shows the geolocations plotted in the ecological maps of the state of Tripura, India. The area where the app was deployed was rural and a densely forested region where malaria surveillance was remotely monitored and tracked in real time.

To assess the functionality of the app and its preference over a paper-based system, a survey was conducted among 19 health care workers using the app via Google Forms in English and 2 local languages (Bengali and Kokborok). The survey showed that all the users found the app easy to use and learn, with an average of 2 to 3 minutes per entry, and the users preferred to continue using the app over the paper-based system (Figure 5B). The MPWs emphasized that SMS text messaging features of the app are most useful as SMS text messages are received when an ASHA or a village volunteer reports a malaria patient during an active surveillance in the village, in addition to the status of the treatment administered. This prevents delays in the actions to be taken by MPWs. Furthermore, the local language and pictorial options make it easy and lucrative to be used by the ASHA and village volunteers. During paper-based recording of malaria cases, the translation of surveillance data from the local language to English is difficult and often results in delayed reporting. The additional feature of the village household database ensures that the data are verified, and no typing or data entry errors occur during data collection and further processing from local languages to English.

The health care workers, ASHAs, and village volunteers further emphasized that the minimal typing feature and the availability of the app in the local language made it easy and lucrative over paper-based reporting. Furthermore, the pictorial drug information in the app ensured an accurate dosage of drugs administered to the patients. The offline data capture feature of the app will be useful in remote areas of certain parts of India with no or poor connectivity. The use of the app has reduced the time delay from the collection of data to their reporting to the district or state health care system by over a month owing to the instantaneous SMS text message delivery system, which has made all concerned people in health systems aware of any positive case detection in the study area. The app was useful in these 19 remote villages with long electricity power cut sessions, which were overcome by supplying power banks for charging the mobile handsets.

## Discussion

### Principal Findings

The FeverTracker app was deployed in 19 malaria-endemic tribal villages situated in remote, poorly networked, hilly, forested regions of the state of Tripura in India via health care workers, ASHAs, and village volunteers. Over a period of 20 months, the use of the app resulted in regular data collection, shortening the time between data collection and processing by the health workers by over a month. According to a survey conducted for app users, FeverTracker was reportedly easy to use, beneficial for drug guidance, took less time to learn and use, reduced errors, and helped in village-level line listing with instant communication to the health system.

### Strength and Limitations

The FeverTracker app has been particularly useful for malaria surveillance during the COVID-19 period with restricted mobility. The app records a range of fever-related symptoms, including COVID-19. This allowed the village volunteers and ASHAs to survey their respected villages, and the data collected reached the MPWs and district or state health officials without requiring the physical movement in or out of these villages. At the core of malaria management is extensive and coordinated surveillance of the community by health care workers at multiple levels. Among the challenges faced, limited mobility and resources along with the high number of target populations for surveillance compromises coverage and epidemiological data collection. Thus, the app offers a timely solution to these issues.

The app as a data collection tool, with minimal typing, pictorial, and automatic digitization features, reduces the time, effort, and resources that are presently dedicated toward paper-based collection and digitization. This app also reduces the major current limitation of the mobility or physical travel of surveillance teams after the completion of the survey to provide updated data to the concerned health professionals. As a result, the entire process of data capture to analysis will be hastened, thus enabling rapid decision-making on the mitigation steps needed. FeverTracker has been designed such that it can be used by both private health care providers and the community to report malaria treatments undertaken at a private health care facility. Furthermore, the app will increase health seeking by the community as they would be self-reporting on suspicion of symptoms, preventing delays and other inconveniences associated with seeking the attention of a health care worker by shortening the channel of communication between the patient and the malaria health care personnel. These features of the FeverTracker app allow identifying and tracking of a vast number of patients outside the radar of malaria surveillance of the government and add robustness to the collection of epidemiological parameters required to track the progress of malaria control and elimination with the help of both the community and the health workers.

### Conclusions

Currently, mobile health apps are being used to assist in monitoring patients outside hospitals, track vitals, or analyze medical images for physicians. More recently, the Food and Drug Administration started to approve health apps and devices [16]. Furthermore, the COVID-19 pandemic has shown the world utility of app-based data collection systems that are useful not only in prompt reporting, and hence data-based decisions, but also in creating awareness and alertness among communities [17,18]. In this light, FeverTracker has the potential to provide holistic support in the management of malaria control. It can also be used for joint surveillance and control programs of other eliminable vector-borne diseases, such as visceral leishmaniasis and lymphatic filariasis, which is desirable for the country at this juncture [19]. In sum, this app will allow (1) the omission of paper-based data capture (symptoms, test results, and medicines prescribed), (2) real-time communication of data to district and state surveillance teams, (3) pictorial representation and local language support for ground-level health workers, (4) offline data capture, (5) geotagging, and (6) link-up with web application–based dashboards that will allow the health authorities to locate transmission foci and hotspots. The app has been specifically designed, so it can be tailored according to the requirements of the government programs of any state or country. A gradual switch to app-based malaria surveillance will improve coverage, case data integration, data analysis, and epidemiological visualization and will inform malaria control programs in near real time. This robust *FeverTracker* app can be integrated within the health systems at the district, state, or national level to build a health care system network and can also be used for tracking acute fever or febrile illness other than malaria, such as dengue, leptospirosis, influenza, influenza A, rickettsial infections, Japanese encephalitis, and chikungunya and other infectious diseases, including COVID-19, while providing a long-term support to the health care system and public health research in the country.

## Abbreviations

ASHA: Accredited Social Health Activists
ACD: active case detection
GIS: geographic information system
MPW: multipurpose worker
NVBDCP: National Vector Borne Disease Control Programme
PCD: passive case detection
PHP: hypertext preprocessor
RDT: rapid diagnostic test
WHO: World Health Organization
